# Disseminated TB associated with acute severe malnutrition in a Roma child

**DOI:** 10.5588/ijtldopen.24.0454

**Published:** 2024-12-01

**Authors:** P. Kunč, J. Fábry, P. Ferenc, T. Strachan, M. Matiščáková, I. Solovič, R. Péčová, M. Dohál

**Affiliations:** ^1^ Clinic of Pediatric Respiratory Diseases and Tuberculosis/National Institute of Pediatric Tuberculosis and Respiratory Diseases, Dolny Smokovec Comenius University, Jessenius Faculty of Medicine in Martin, Bratislava, Slovakia;; ^2^ Department of Pathological Physiology, Comenius University in Bratislava, Jessenius Faculty of Medicine in Martin, Bratislava, Slovakia;; ^3^ National Institute of Tuberculosis, Lung Diseases and Thoracic Surgery, Vyšné Hágy, Slovakia; Faculty of Health, Catholic University, Ružomberok, Slovakia;; ^4^ Biomedical Centre Martin, Jessenius Faculty of Medicine in Martin, Comenius University, Bratislava, Slovakia.

**Keywords:** severe tuberculosis, acute malnutrition, secondary immune deficiency, Roma ethnicity, paediatric tuberculosis

Dear Editor,

TB is an infectious granulomatous disease caused by *Mycobacterium tuberculosis* (MTB) and remains a global health challenge. The paediatric population exhibits unique characteristics in relation to TB infection, and although they represent only 10% of global TB notifications, children experience a significantly higher mortality rate than adults. Despite a decrease in reported childhood TB cases in Western and Central Europe, a substantial number of cases continue to emerge from marginalised communities. Here, we report on the onset of severe TB in a Roma child from a socially deprived background who showed signs of acute severe malnutrition.

**Figure 1.**
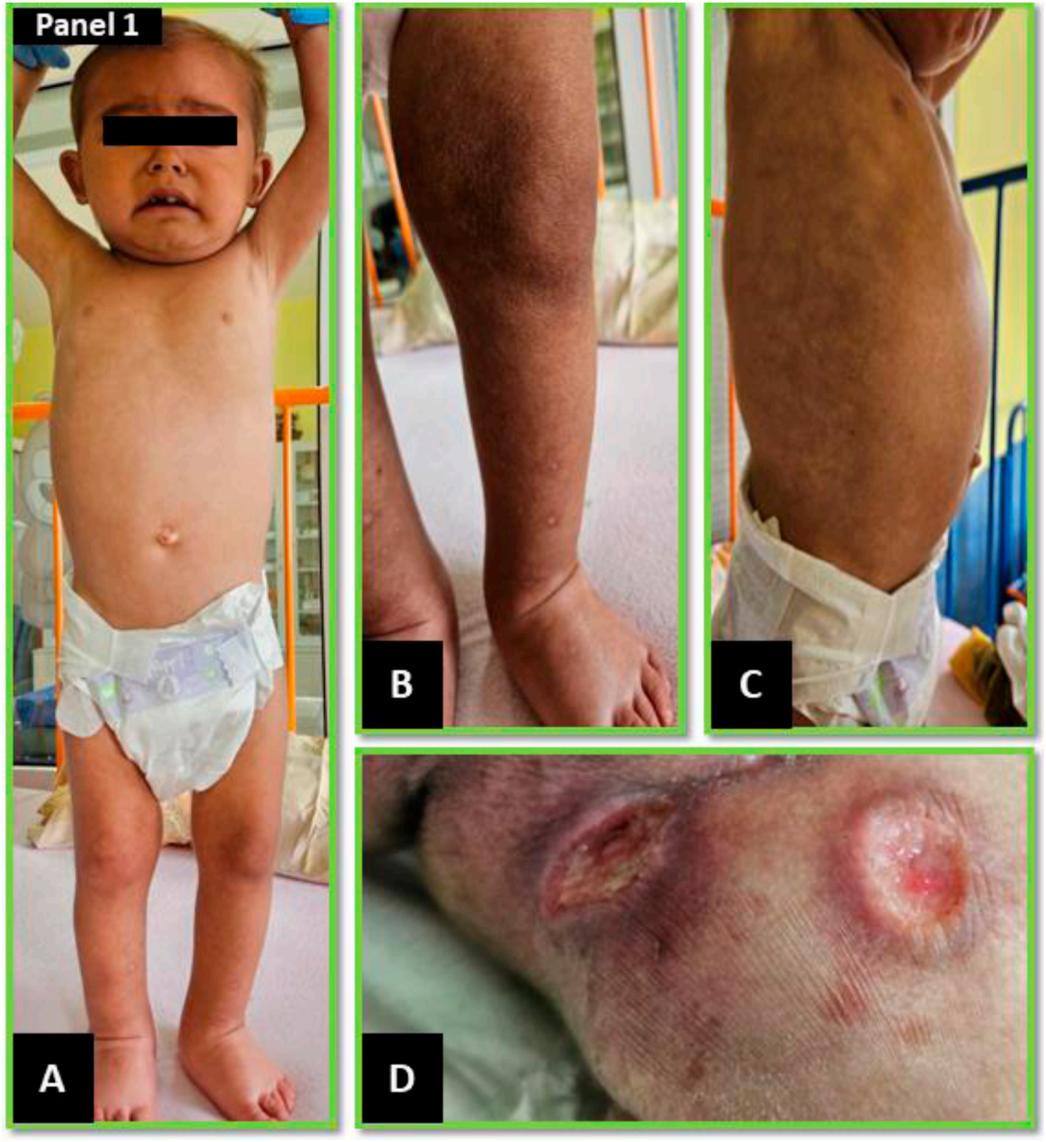
**A** and **B)** Anasarca mainly on the lower limbs and dorsum of the feet; **C)** abdominal distension in the sagittal plane; and **D)** lymph node bundles in the inguinal region with fistulation and formation of characteristic skin defects before treatment.

**Figure 2.**
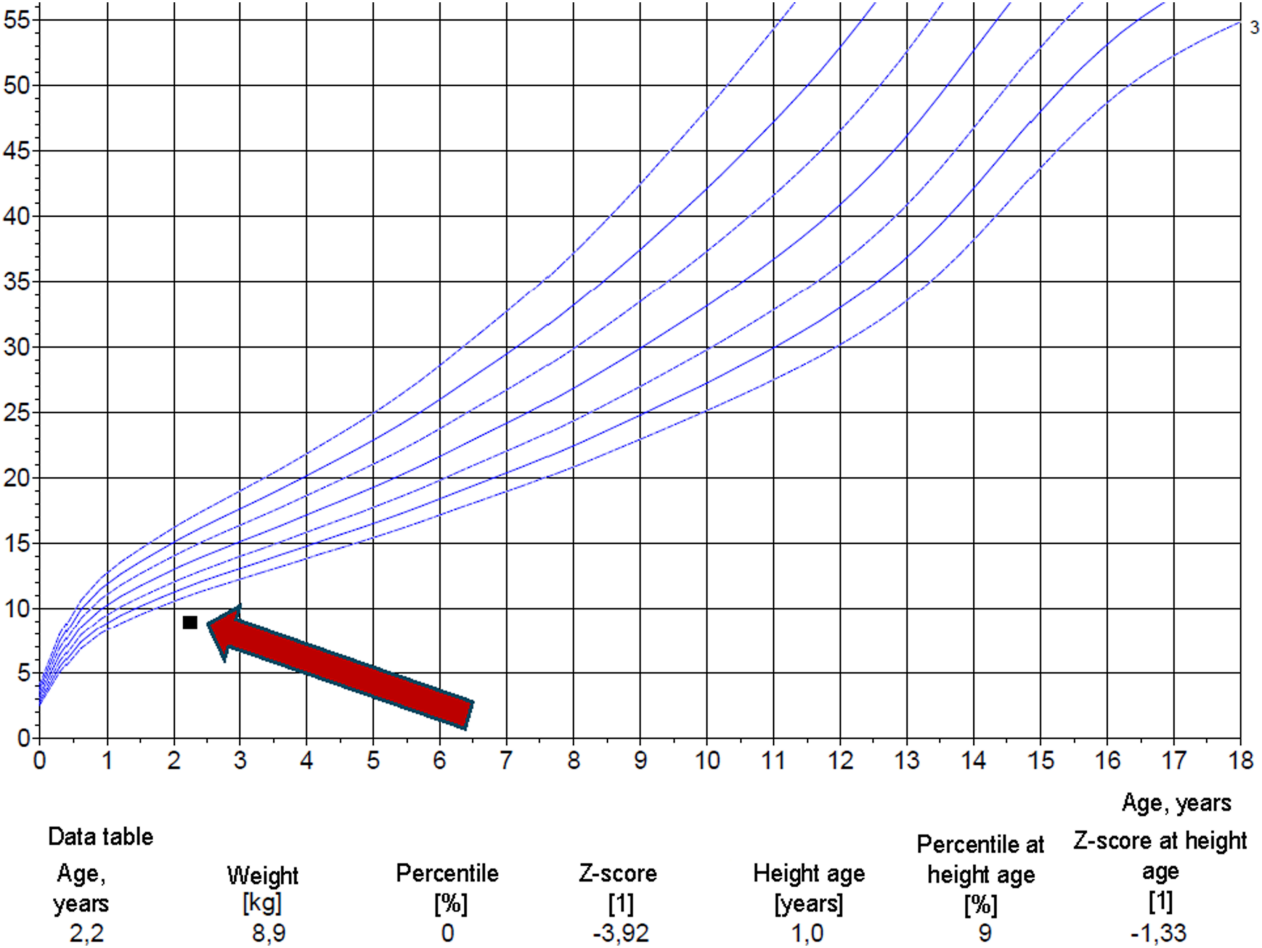
Growth diagram.

A 26-month-old Roma male child not vaccinated with bacille Calmette-Guerin (BCG) was admitted to the regional paediatric ward due to generalised lymphadenopathy. Being from a socially deprived environment characteristic of marginalised communities, the home lacked running water and proper sanitation, with solid fuel heating and passive exposure to tobacco smoke. The child presented generalised skin and subcutaneous tissue oedema, most pronounced in the lower extremities and dorsal region of the feet (Figure 1A, B). Distention of the abdomen was evident in the lateral profile (Figure 1C). The cervical and inguinal lymph nodes were enlarged. Two painful skin ulcerations with a whitish base and serous exudate were present in the left inguinal region (Figure 1D). Anthropometric measurements revealed significant growth retardation. The child’s weight was markedly below the third percentile for age (*Z* score –3.92) (Figure 2), and height corresponded to a 1-year-old. Subsequent chest computed tomography revealed a miliary pattern of small nodules within the lung parenchyma, accompanied by mediastinal and perihilar lymphadenopathy. Ultrasonography revealed caseous necrosis of lymph nodes with incipient calcification and fistulation in the neck and inguinal region. Similar findings in the splanchnic lymph nodes were consistent with tuberculous enteritis and mild paracolic ascites, suggesting disseminated gastrointestinal TB. An interferon-gamma release assay (IGRA) test (QuantiFERON TB-Gold Plus; Qiagen, Hilden, Germany) yielded a positive result. The patient was urgently initiated on treatment with a regimen of first-line anti-TB drugs comprising isoniazid, rifampicin, pyrazinamide and ethambutol, supplemented with symptomatic therapy according to clinical standards.^1^ Laboratory evaluation revealed a secondary cellular immunodeficiency linked to severe malnutrition. Flow cytometry immunophenotyping demonstrated a disruption of the lymphocyte subset, characterised by a marked reduction in cytotoxic CD8+ lymphocytes (1,333/µl) relative to CD4+ lymphocytes (4,240/µl). This imbalance and an elevated immunoregulatory index (3.18) indicated potential immune dysregulation. Furthermore, a decrease in total monocyte count (CD14+, CD16±; 430/µl) with a predominance of classical monocytes (CD14 + 16–) suggested a heavy bacterial load and the risk of lymphocyte suppression as a compensatory anti-inflammatory response. Targeted nutritional support was initiated due to the severe acute energy protein malnutrition, characterised by marked hypoalbuminemia (19 g/L) and near-absent pre-albumin (0.06 g/L). Molecular biological methods (polymerase chain reaction) confirmed the presence of MTB in gastric content aspirate and skin ulceration swabs from the inguinal region. MTB was microbiologically cultivated after 3 weeks post-collection from gastric juice. Microscopic examination of the gastric aspirate was also positive. Whole-genome sequencing (WGS) was employed to characterise the MTB strain comprehensively. Sequencing data analysis revealed the strain’s susceptibility to all first- and second-line anti-TB drugs. However, the strain carried a mutation in the *rps*A (Pro163Ser) gene and deletion in Rv3083 (1407del) gene, linked to resistance to PZA and ethionamide, whose clinical significance remains unclear. Furthermore, the strain belonged to the Euro-American phylogenetic sublineage 4.8. Legal guardians provided written informed consent prior to their child’s participation in the study.

Global notification rates for TB are unevenly distributed, with sub-Saharan Africa (30%) and South-East Asia (48%) carrying the heaviest load. These regions also report alarmingly high TB mortality rates, affecting both HIV-positive and -negative children (up to 84%).^2^ In contrast, Slovakia has witnessed a gradual decline in childhood TB incidence: data from the European Centre for Disease Prevention and Control (Stockholm, Sweden) indicate a decrease in TB prevalence among children from 5.1/100,000 in 2017 to 4.7/100,000 in 2021.^3^ However, this trend was disrupted in 2022 due to the armed conflict in Ukraine. Slovakia discontinued universal BCG vaccination in 2012 mainly due to a significant reduction in TB notifications.^4^ We can assume with reasonable confidence that BCG vaccination of the child would have protected against the condition described above. TB is a social disease that predominantly affects marginalised populations living in poverty, including the Roma community. In Slovakia, Roma ethnicity constitutes approximately 8–10% of the population and is over-represented among childhood TB cases. A similar trend occurs in the countries of Central Europe.^5^ Recent data from the Czech Republic indicate that almost one-third of children with TB are of Roma ethnicity.^6^ Given Slovakia’s large Roma population compared to the Czech Republic, it is likely that the incidence of childhood TB is even higher. Multiple factors contribute to the rapid spread of TB within Roma communities, including generational poverty, low educational attainment, smoking, stigmatisation of the disease, and distrust of healthcare systems.^7^ This lifestyle, in combination with other identified risk factors, contributes significantly to the burden of TB in the paediatric population. As close adult contacts are the primary source of infection for children, these circumstances pose a substantial challenge for TB prevention and control in this vulnerable group. In conjunction with severe malnutrition, TB constitutes a major cause of mortality, particularly among impoverished children under five years of age. This age group is highly susceptible to the progression of primary TB into disseminated forms, with a significantly increased risk of death if treatment is not administered promptly and adequately.^8^ In its efforts to eliminate childhood TB, the WHO emphasises the bidirectional relationship between malnutrition and TB as a critical challenge, especially in low-to middle-income countries.^9,10^ Severe malnutrition, a global health problem, exacerbates this vulnerability by inducing secondary immunodeficiency and also affects the pharmacokinetics of anti-TB drugs.^11^ In children with TB and malnutrition, decreased expression of pro-inflammatory T-helper 1 (Th1) modulated cytokines (interleukin [IL]12, IL-18, IL-21) has been observed, with impaired Th1 lymphocyte differentiation and maturation reducing IL-2 and interferon-gamma (IFN-γ) production.^12^ Given the impaired immune response to MTB in malnourished children, strategies are emerging to modulate and restore immune balance. The phylogenetic lineage of MTB also significantly impacts a patient’s clinical presentation and immunological response.^13,14^ The strain identified here was classified as Euro-American Lineage 4 based on phylogenetic analysis. Notably, this lineage has been associated with a lower risk of disseminated TB due to an *EsxM* gene stop codon, as reported in a recent study.^15^ Although phylogenotyping indicated a low-risk MTB strain, severe acute malnutrition likely played a substantial role in the widespread dissemination.

Our case report highlights the urgent need for comprehensive TB control strategies, particularly in vulnerable populations of industrialised countries.
